# Cardiac Rehabilitation Facebook Intervention: Feasibility Randomized Controlled Trial

**DOI:** 10.2196/46828

**Published:** 2023-06-15

**Authors:** Lee Anne Siegmund, James F Bena, Shannon L Morrison

**Affiliations:** 1 Nursing Research and Innovation Cleveland Clinic Cleveland, OH United States; 2 Quantitative Health Sciences Cleveland Clinic Cleveland, OH United States

**Keywords:** cardiac rehabilitation, motivation, exercise, social media, cardiology, adherence, physical activity, satisfaction, rehabilitation, Facebook, peer support

## Abstract

**Background:**

The adherence to cardiac rehabilitation is low. Social media has been used to improve motivation and cardiac rehabilitation completion, but the authors did not find Facebook interventions for these purposes in the literature.

**Objective:**

The purpose of this study was to determine the feasibility of the Cardiac Rehabilitation Facebook Intervention (Chat) for affecting changes in exercise motivation and need satisfaction and adherence to cardiac rehabilitation.

**Methods:**

The Behavioral Regulation in Exercise Questionnaire-3 and Psychological Need Satisfaction for Exercise were used to measure motivation and need satisfaction (competence, autonomy, and relatedness) before and after the Chat intervention. To support need satisfaction, the intervention included educational posts, supportive posts, and interaction with peers. The feasibility measures included recruitment, engagement, and acceptability. Groups were compared using analysis of variance and Kruskal-Wallis tests. Paired *t* tests were used to assess motivation and need satisfaction change, and Pearson or Spearman correlations were used for continuous variables.

**Results:**

A total of 32 participants were lost to follow-up and 22 were included in the analysis. Higher motivation at intake (relative autonomy index 0.53, 95% CI 0.14-0.78; *P*=.01) and change in need satisfaction-autonomy (relative autonomy index 0.61, 95% CI 0.09-0.87; *P*=.02) were associated with more completed sessions. No between-group differences were found. Engagement included “likes” (n=210) and “hits” (n=157). For acceptability, mean scores on a 1 (not at all) to 5 (quite a bit) Likert scale for feeling supported and in touch with providers were 4.6 and 4.4, respectively.

**Conclusions:**

Acceptability of the Chat group was high; however, intervention feasibility could not be determined due to the small sample size. Those with greater motivation at intake completed more sessions, indicating its importance in cardiac rehabilitation completion. Despite challenges with recruitment and engagement, important lessons were learned.

**Trial Registration:**

ClinicalTrials.gov NCT02971813; https://clinicaltrials.gov/ct2/show/NCT02971813

**International Registered Report Identifier (IRRID):**

RR2-10.2196/resprot.7554

## Introduction

### Background

Cardiac rehabilitation (CR) is an evidence-based clinical standard due to reported benefits for functional capacity, psychosocial health, and mortality [1]. Cardiac rehabilitation referral is a class IA recommendation by the American Heart Association and American College of Cardiology for patients with recent myocardial infarction [2], coronary artery bypass graft surgery [3], and percutaneous coronary intervention [4]. The Centers for Medicare and Medicaid Services have also approved CR for patients who had heart valve repair or replacement or those who had heart or heart and lung transplant [5]. Additionally, patients with heart failure can benefit from outpatient exercise-based CR (phase II), which has been shown to improve oxygen uptake, muscle health, and left ventricular ejection fraction in this population [1]. Despite these and other benefits, uptake, adherence, and completion of 12-week phase II CR programs remain poor.

### Cardiac Rehabilitation Adherence

Cardiac rehabilitation referral rates vary by location; however, many hospitals automatically refer all patients with a qualifying diagnosis [6]. Nevertheless, researchers continue to report numerous barriers to CR uptake and adherence, and only 34% of those who are referred actually attend CR [7]. For many, barriers are access related and include cost and lack of transportation [8,9]. Patients may also experience disparate access related to gender, race, ethnicity, and poor physical health [9]. Unfortunately, solutions addressing barriers have not always been effective; however, low-cost approaches have been recommended [9]. Supervia et al [10] reported that relationships with CR staff and other patients were important to those who completed the program. Other researchers described that, for women, motivation to complete the program included a friendly attitude and reinforcement by the CR staff [11]. These patients completed the program despite encountering multiple barriers [11], suggesting that support from the staff may have been an important factor in their program completion.

### Technology-Driven Solutions

For patients who begin CR, technology-driven solutions may be helpful for providing additional support and reinforcement to ensure completion [12]. For example, patients attended more sessions when they used a mobile technology app that tracked their metrics, provided educational materials, and enabled communication with the CR team compared to patients in usual care CR [13]. Researchers also showed that patients from low-income households found social media platforms acceptable for targeting lifestyle changes [14]. In China, a WeChat social media intervention combined with a 12-week center-based CR program was successful at improving adherence and completion, with the intervention group attending at least 75% of sessions [15]. Additionally, perceived health scores and 6-minute walk test improved [15]. Motivational factors may play a role in technology-supported CR. In a comparison between telerehabilitation (home-based) and traditional center-based CR, perceived competence increased in both groups, and the telerehabilitation group had an initial increase in motivation; however, there was no difference in motivation over the long term, demonstrating that a more intense intervention might be needed for motivation to be internalized [16].

No Facebook interventions for cardiac rehabilitation adherence were found in the literature. Nevertheless, patients with greater Facebook capability were receptive to the idea of using the platform for a closed CR group and peer support [17]. Other researchers posited that applications such as Facebook may be valuable CR tools to enhance social connectedness [18]. Since Facebook is free and widely used (70% of American adults report regular and often daily use) [19], it may be a promising supplement to CR for the purposes of information sharing and support, with the potential to improve motivation for exercise and CR adherence.

### Theoretical Framework

**Figure 1:**
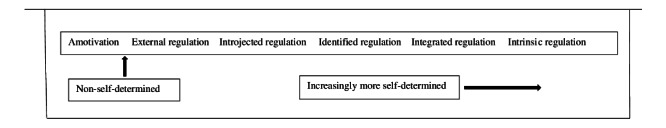
Self-determination theory and motivation continuum.

The Cardiac Rehabilitation Facebook Intervention (Chat) was grounded in self-determination theory (Figure 1). Self-determination theory was successfully used in prior CR research that used pedometers to support self-determined motivation [20]. Further, researchers investigated self-determination theory in a content analysis on Facebook in older adults [21]. According to the theory, motivation is on a continuum, ranging from amotivation (no intention to do the activity) to intrinsic motivation (may do the activity just for the joy of doing it) [22]. According to Ryan and Deci [22], to achieve self-determined motivation (internalized), 3 psychological needs must be met: competence, autonomy, and relatedness [21]. Competence can be supported through structure, positive feedback, and realistic goals [23,24]. Autonomy can be supported by assisting with decision-making for personal reasons and providing minimal pressure to help with making choices [23-25]. Relatedness can be supported by helping a person feel socially included [22,24,26].

### Purpose

The primary purpose of this study was to determine the feasibility of the Chat Facebook intervention, providing education, peer support, and provider support, for affecting change in motivation and self-determination for exercise, and adherence to CR in patients with heart disease during a 12-week phase II CR program compared to a control group who received educational handouts and emails. Feasibility was predicated on the effectiveness of the recruitment strategy, the success of the intervention, participant engagement (number of visits to the group, “hits,” and “likes”), and acceptability of intervention (comments). We hypothesized that Chat would improve motivation for exercise, and participants in the Chat intervention would complete a higher percentage of CR sessions compared to the control. Additionally, we hypothesized that engagement in Chat would predict a higher percentage of CR sessions and would provide evidence of feasibility. Feasibility was also determined by recruitment or sample size and retention.

## Methods

### Design

This was a randomized controlled pilot trial to evaluate the feasibility of using Chat to affect change in motivation for exercise and adherence to cardiac rehabilitation.

### Ethics Approval

This feasibility study was approved as minimal risk research by the hospital’s institutional review board (IRB) (16-1456) and was registered with ClinicalTrials.gov (NCT02971813). Written informed consent was obtained, and each participant was adequately informed about the research. All participants included in the study voluntarily agreed to participate. The consent document included details on randomization to groups, surveys, and participation in the Chat group or the control. Possible risks included in the consent were loss of personal information, computer viruses, and malicious software, as well as steps the researchers would take to minimize these risks. All postings, data collection, storage, and analyses were conducted using a password-protected hospital computer in the locked office of the principal investigator. Participants were also informed that they could withdraw from the study at any time. No compensation was provided, and participants incurred no cost for participating in the study.

### Sample and Setting

A sample size of 30 per group was chosen in order to facilitate a large intervention group since the value of the intervention depended on the interaction among the participants. The researchers conducted an a priori power analysis and determined that an estimated sample size of 60 total participants for randomization would provide 86% power to detect large effect sizes (Cohen *d*=0.8) for the outcomes of interest. We determined that feasibility would be unlikely if a sample of 30 per group could not be obtained in the first year. Participants qualified for participation if they planned to attend the outpatient CR (center-based program) at the main campus and 3 regional hospitals of a large hospital system in Ohio. This 36-session phase II CR program was exercise based and included electrocardiogram-monitored and supervised exercise, and education on diet, stress reduction, and behavioral counseling.

Participants also qualified for the study if they were regular Facebook users (defined as logging in at least twice per month), had a cardiac diagnosis, and had been referred to the cardiac rehabilitation program at the hospital’s main campus or 3 of the regional campuses. Regular use was defined as logging into the Chat group at least 2 times in the last month. Other inclusion criteria included adults who were age 18 years or older, were able to read and speak English, and lived within 100 miles of the cardiac rehabilitation center at the hospital’s main campus. No other exclusions applied.

### Recruitment and Randomization

Recruitment took place during patients’ inpatient stay, intake visit for cardiac rehabilitation, or via phone call within a week after hospital discharge. In order to ensure there were participants with whom to interact in the Chat group, the first 8 volunteers were allocated to Chat, and no data were collected for these participants. After the first 8, participants were randomized to the Chat versus the control groups using blocked randomization, an appointment was scheduled to discuss the study and obtain consent. For convenience, this appointment often occurred just prior to their first CR session, in a private space near the exercise room, and was always at a date and time prior to beginning the CR program. Written informed consent was obtained from each participant. Participants were randomized at the time of consent. As described in the published protocol, randomization was to take place after consent and completion of preintervention questionnaires [27]. However, the extra time required to fill the questionnaires interfered with the start of their first CR visit, so this was changed with the IRB amendment so that participants were randomized and then given the questionnaires via email. A link was emailed to the participants and included the baseline Behavioral Regulation in Exercise Questionnaire-3 (BREQ-3) [28], Psychological Need Satisfaction for Exercise (PNSE) scale [25], and instructions for joining the private Chat group if applicable. The control group was given the same baseline questionnaires and was informed they would be receiving weekly emails. They were also given the opportunity to join the Facebook group after CR completion.

### Study Procedures

#### Overview

Study procedures were described previously in the published protocol [27]. The principal investigator independently completed all recruitment, consent, administration of questionnaires, and intervention implementation. The CR team regularly handed out study flyers to inpatients who were in phase I CR.

#### Intervention Group

Chat was designed to promote increasingly higher self-determined motivation. The intervention, which was participation in the Chat group, included educational posts (to support competence), supportive provider posts (to support autonomy), and opportunities to interact with peers (to support relatedness). Supporting competence, which refers to self-efficacy in learning new information, requires the provision of structure in the information provided, offering participants positive feedback, and helping them to set realistic goals [23-25]. Educational posts were created to support competence and covered 12 topics, offering a variety of suggestions and encouragement for making personal health care choices. Provider posts included topics such as motivational quotes, contained reminders to exercise independently, and provided support for individual decision-making with minimal pressure [23-25]. Peer interaction in the Chat group served to support relatedness and took place as frequently as the participants freely chose to do so. Situations that are conducive to social interaction with peers can help facilitate intrinsic motivation [22]. The educational and provider posts were standardized such that they contained a consistent message for each topic. Content was then posted on the Chat group, 1 each week, then reposted again every 12 weeks to account for rolling enrollment. Per IRB request, all postings were IRB approved prior to being posted on the Chat group.

#### Control Group

The control group received the same educational and provider support materials as the intervention group but in the form of an email. Like the Chat group, the control group participated in the same 12-week phase II CR and received the usual care and education through the CR program.

### Measures and Outcomes

#### Overview

The outcomes for this study were (1) change in motivation for exercise, (2) change in self-determination for exercise (competence, autonomy, and relatedness), (3) adherence to the 12-week CR program, and (4) measures of feasibility (recruitment strategy, success of the intervention, engagement, and acceptability).

#### Engagement

Engagement, defined as participation in the Chat group, was determined by the number of “likes” and “hits” in the group. “Likes” (the number of times a participant clicked “like” on any of the Facebook posts) were counted. The number of “hits” was self-report using a postintervention questionnaire.

#### Acceptability

Acceptability of the intervention was determined from a post questionnaire, which had a section for additional comments. The post questionnaire also included questions about feeling supported, changing behaviors, and feeling healthier as a result of participation in the Chat group. Responses were on a 5-point Likert scale (1: “not at all” to 5: “quite a bit”).

#### Participant Characteristics

Participant characteristics were collected from the electronic medical record and included age, sex, race, and diagnosis. In addition, intake functional capacity was collected. Functional capacity was measured in metabolic equivalents and obtained from the intake stress test prior to CR.

#### Motivation

Change in motivation for exercise was measured using the BREQ-3, a 24-question instrument based on self-determination theory that measures intrinsic and extrinsic regulation of exercise behavior [28]. Cronbach α reliabilities for the BREQ-3 subscales (regulations) were: amotivation (.83), external regulation (.79), introjected regulation (.80), identified regulation (.73), and intrinsic regulation (.86). Each question in the BREQ-3 can then be weighted (ranging from −3 to +3) and summed, giving a single score of self-determination for exercise known as a relative autonomy index (RAI). The RAI is useful for determining an individual’s motivational subtype (behavioral regulation) from amotivated (lacking intention to exercise) to intrinsically motivated (self-determined or autonomously motivated).

#### Psychological Need Satisfaction

Perception of psychological need satisfaction was measured with the PNSE to determine the extent to which participating in exercise promoted feelings of competence, autonomy, and relatedness, which are the 3 subscales of the PNSE [25]. The PNSE consists of 18 items on a 6-point Likert scale from 1 (false) to 6 (true). Higher scores for each latent factor indicate higher competence (eg, I feel confident in my ability to perform exercises that personally challenge me), autonomy (eg, I feel like I am the one who decides what exercises I do), or relatedness (eg, I feel connected to the people who I interact with while we exercise together). Cronbach α was >.90 [25]. Normative data, collected from a sample of Canadian university exercise class participants, are also available [29]. Adherence to the CR program was reported as a percentage of cardiac rehabilitation sessions attended and was measured by dividing the number of sessions attended in a 12-week period by the total number of sessions allowed by insurance and multiplying by 100.

### Analysis

Categorical variables were described using frequencies and percentages, and analyses comparing the control and the Chat groups used Pearson chi-square or Fisher exact tests. Normally distributed continuous variables were described using means and SDs, and analyses comparing control and Chat groups used analysis of variance models. Nonnormally distributed continuous variables were described using medians and quartiles, and analyses comparing the control and the Chat groups used Kruskal-Wallis tests. Paired *t*-tests were used to assess RAI and PNSE change within groups. The relationship between RAI change and continuous variables was assessed using Pearson or Spearman correlations (for the number of sessions, which was not normally distributed) with 95% CI. For categorical measures, means and SDs with *P* values from analysis of variance models. The relationships between the number of sessions with continuous variables were assessed using Spearman correlations with 95% CI, while medians and quartiles are presented for categorical factors. Internal consistency was determined with Cronbach α. Analyses were performed using SAS software (version 9.4; SAS Institute, Inc). A significance level of .05 was assumed for all tests.

## Results

### Recruitment and Participants

**Figure 2:**
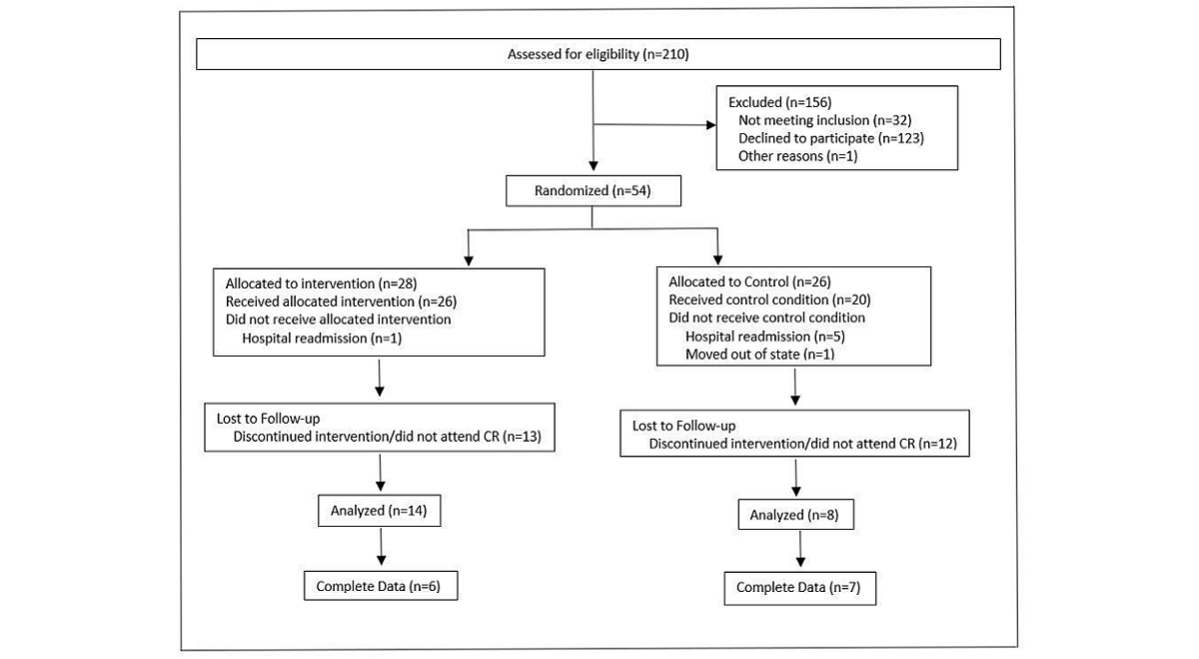
Consort diagram for recruitment. CR: cardiac rehabilitation.

After removing those who were participating in CR for noncardiovascular disease–related reasons, 210 potential participants were screened for eligibility. Of 54 (26%) who agreed to take part in the study, 28 were randomized to intervention and 26 to the control group. See Table 1 and Figure 2 for additional recruitment and participant characteristics. There were no differences between groups on personal characteristics at baseline. Of the final analyzed sample, diagnoses included aortic aneurysm repair (n=2), myocardial infarction (MI) (n=7), coronary artery bypass graft without MI (n=1), percutaneous coronary intervention without MI (n=1), Takotsubo’s cardiomyopathy (n=1), valve repair or replacement (n=3), heart transplant (n=2), and other (n=5). Of additional participant medical conditions, 64% had hypertension, 14% had diabetes, 14% had hypertriglyceridemia (>150 mg/dL), 32% had elevated low-density lipoprotein (≥100 mg/dL), and 45% had low high-density lipoprotein (<60 mg/dL). The mean functional capacity for participants at intake to CR was 5.9 (SD 2.4) METS. Data for exit metabolic equivalents were not analyzed as they were only available for 3 participants. Of the 14 remaining in the intervention group, 6 (43%) completed the exit questionnaires compared to 7 (87%) in the control group.

**Table 1:** The relationship between group and demographic variables.

Factor			Overall (N=22)		Control (n=8)		Facebook (n=14)		*P* value	
Age (years), mean (SD)			57.8 (11.0)		60.6 (7.2)		56.2 (12.6)		.38^a^	
**Sex, n (%)**										.19^b^
	Male	9 (41)		5 (62)		4 (29)				
	Female	13 (59)		3 (37)		10 (71)				
**Race, n (%)^c^**										.99^b^
	Black	6 (28.6)		2 (25)		4 (31)				
	White	15 (71)		6 (75)		9 (69)				
RAI^d^ intake, mean (SD)			39.4 (23.5)		32.9 (14.9)		43.1 (27.0)		.34^a^	
Intake METS^e,f^			5.9 (2.4)		6.5 (2.2)		5.6 (2.6)		.52^a^	
**Diagnosis categories, n (%)**										.08^g^
	CAD^h^	14 (64)		7 (87)		7 (50)				
	Other	8 (36)		1 (12)		7 (50)				
CR^i^ sessions out of 36, median (IQR)			26.0 (4.0-36.0)		22.0 (3.5-27.0)		32.5 (10.0-36.0)		.21^j^	

^a^Analysis of variance.

^b^Fisher exact test.

^c^Race was reported for 8 participants in the control and 13 in the Facebook group, for a total of 21.

^d^RAI: relative autonomy index.

^e^METS: metabolic equivalents.

^f^Intake METS was reported for 6 participants in the control and 10 in the Facebook group, for a total of 16.

^g^Pearson chi-square test.

^h^CAD: coronary artery disease.

^i^CR: cardiac rehabilitation.

^j^Kruskal-Wallis test.

### Intervention

Across both groups, higher motivation (RAI) at intake was associated (95% CI) with the greater number of completed sessions (RAI 0.53, 95% CI 0.14-0.78; *P*=.01). Change in PNSE need satisfaction-autonomy from pre-to-post intervention was also associated with a higher number of completed sessions (RAI 0.61, 95% CI 0.09-0.87; *P*=.02). No between-group differences were found for change in motivation, need satisfaction, or sessions completed. There was no relationship between demographic variables and change in motivation, need satisfaction, or sessions completed.

### Engagement

There were 210 “likes” and 157 “hits” in the Chat group. Comments in the Chat group (n=21) were positive (86%) and the other 14% of comments were neutral. Exit questionnaires for the Chat group indicated that 4 participants logged into the group (hits) a total of only 1-5 times, one logged in 6-10 times, and 1 participant never logged in to the group.

### Acceptability

Comments on the post questionnaire included “Very interesting, informative and educational” and “I loved the videos.” Some commented specifically on the cardiac rehabilitation sessions. These statements included: “I thoroughly enjoyed being with my group in cardiac rehab!” “We cared about each other and laughed together, as we worked hard at completing our exercise programs,” “I love going,” and “The staff was wonderful!” For those who dropped out of CR, statements included: “I prefer to do it on my own,” “I just don’t have the time,” and “I’m overwhelmed with my health and all of my appointments.”

The mean response for feeling supported by the Chat group and feeling more in touch with their CR health care team was 4.6 (SD 0.89) and 4.4 (SD 0.89), respectively, both with a range of 3-5 on a 5-point Likert scale. When asked how often they talked with other Chat members in the web-based group, responses ranged from “not at all” to “rarely” except for one participant who stated “frequently” (mean 2.2, SD 1.64). Mean response to whether they made changes to their exercise or diet after being in the Chat group was 3.4 (SD 1.5) for both, with a range from 1 to 4. The mean for perceptions of overall health as a result of being in the Chat group was 4 (SD 1.4) with a range of 2-5.

## Discussion

### Principal Findings

Despite that recruitment was challenging and we did not meet recruitment goals, there were many positive lessons to be learned from this feasibility study. First, while the sample was small, the Chat intervention was acceptable to the participants based on postintervention feedback. Additionally, there were associations between motivation at intake and greater number of CR sessions, and improvement in perceived competence and a greater number of CR sessions for both groups. No between-group differences were found possibly due to the limited statistical power related to the small sample size. Although no change scores for functional capacity were available due to the lack of exit exercise tests, participants were very poorly conditioned at intake with a mean of 5.9 (SD 2.4) METS, which is very poor for this age group [30]. All of these findings provide helpful information for future research using social media to improve motivation for exercise and CR adherence.

There was a unique opportunity to learn from 2 participants, who were identical twins. During their time in CR, they lived together and provided each other with additional support. They had identical diagnoses, the same surgery at the same time, the same fasting blood sugar, and at intake to CR, functional capacity (metabolic equivalents) was identical. They also scored exactly the same on the pre-RAI, and both had similar positive statements postintervention. However, their homes were far apart. One twin lived out of state, and at the end of her CR sessions, had to return to her out-of-state home. Interestingly, her RAI scores from pre to post went down, while her sister’s scores went up. It may be that the time they had together supported motivation for the out-of-state twin, and since she was not intrinsically motivated at intake and had to stop her sessions 2 weeks before completion, the loss of support accounted for her drop in autonomy scores. A similar phenomenon was cited in the literature, in which researchers posited that a longer intervention time may have been needed to improve motivation for CR participants [16].

### Recruitment

A slowing rate in recruitment coincided with social media privacy issues in national news. For this reason, we chose to continue the study in hopes that recruitment would improve over time. However, 9 months after the start of the study, the research team determined that recruitment was going to be an ongoing issue. It is possible that there was a lack of trust in Facebook or other concerns about privacy that affected the choice to participate or not. It is also possible that potential participants were hesitant to participate due to concerns that they might be randomized to the control group. Only 26% of those who were approached agreed to participate, and of those who were randomized to control, some expressed disappointment, even though they were informed they could join the Facebook group after completing CR. Additionally, 5 in the control group were lost to medical issues at the start, and we were unable to determine if they were later eligible to participate in CR or the study.

Consistent with low CR retention rates nationwide, retention in the Chat intervention was poor. One participant in this study stated that they dropped out of CR and Chat due to feeling overwhelmed with all of their health problems. Others stated that they preferred to exercise on their own or that time was a barrier to CR attendance. Two patients with recent heart transplants encountered medical complications, resulting in CR and study dropout. Previous work revealed that heart failure was associated with a greater likelihood of CR dropout [16], providing further evidence that the sickest patients may have difficulty adhering to the program. It is possible that, given time and support, some of the aforementioned participants would have continued with CR as well as the Chat intervention. Recent reports showed that patients in CR require reassurance, validation, and interaction with staff and other patients in order to complete the program [11]. Therefore, adjustments to the Chat intervention may be needed if it is to provide the required interaction to support CR adherence.

Additionally, motivational support provided via social media platforms could be helpful in post phase II CR programs when patients may be more receptive to additional help and support. Thus, provision of Chat or similar groups should be piloted in phase III and IV programs in which patients are in higher intensity exercise programs and long-term management of heart disease.

### Intervention

Although there were no between-group differences in change in motivation for exercise nor need satisfaction as a result of the Chat intervention, among those in both groups, results showed that those who had an increase in perceived competence were more likely to complete more sessions. Furthermore, those with higher motivation at intake completed more sessions. These findings demonstrate the important role of motivation and competence in CR completion. Nonetheless, we encountered unique issues with the Chat intervention. The IRB determined they had to approve all posts prior to posting. This limited the variety of posts and resulted in a number of IRB amendments. Furthermore, the requirement for IRB prescreening of posts made it difficult for researchers to interact with the participants since any reply that the researchers posted had to be cleared by the IRB first, ultimately limiting communication. These same obstacles prevented the CR staff from posting in the Chat group. In previous research, effective 2-way communication between the patient and CR staff was a vital component of the electronic intervention, which demonstrated improved adherence [13]. Recently, participants in a social media group (WeChat) completed more CR sessions (>75%) compared to the control, and participants were able to regularly communicate with providers through the group’s chat function [15]. Unlike this study in which 2-way communication was limited, the WeChat group was highly interactive [15].

Other researchers suggested that a technology-based intervention was an external motivator [16]. We do not know if this was the case with Chat; only that the intervention was not successful in changing motivation for exercise. A larger study in which participants and researchers can freely communicate may show different results.

### Engagement

The number of “likes” and comments in the group was low. Participants logged into the Chat group rarely and few chatted with other group members. Engagement was likely affected by the limitations of the interventions previously mentioned. Since platforms such as Facebook can provide abundant social connectedness and provider support [18], it is imperative that researchers find ways to engage those who can benefit the most from groups like Chat.

### Acceptability

Overall, the acceptability of the intervention was positive for those who participated. Although there were few comments on the Facebook group, none of them were negative in nature. Acceptability of social media interventions may be higher if individualized for the participant. In a recent systematic review, researchers concluded that a one-size-fits-all approach to social media or other e-based lifestyle interventions is not optimal [14]. Unfortunately, we were not able to individualize the Chat intervention due to IRB restrictions. The post questionnaire revealed that scores were high for “feeling supported” and “feeling in touch with providers”; nevertheless, it is likely that additional support was needed for those who dropped out of CR and the Chat intervention.

### Summary and Future Research

This study provides a number of lessons learned that can be incorporated into the design of future social media research and programming. It is possible the reasons given for poor CR uptake, adherence, and completion are the same reasons for low recruitment, engagement, and postintervention follow-up in the Chat intervention. Similar to this study, lack of time and feeling overwhelmed with health problems were previously reported [10], which reinforces the need for additional support for CR attendees. With the proper support, patients are more likely to attend and complete phase II CR despite obstacles [11]. However, the support provided in Chat may have been more effective with an alternative social media platform and methodology to improve the interaction between participants and providers.

### Recruitment

Recruitment was suboptimal in this study and might be improved in future research. First, recruiting participants from Facebook, rather than from a limited cohort within a single hospital system, might offer the benefit of sampling from a large number of volunteers. While recruiting from Facebook is more likely to bias the sample toward those who have strong feelings or motivation, it allows for a larger and more diverse group of volunteers. It is possible that recruiting from multiple hospital systems while also including those who have never been on Facebook might increase recruitment numbers but would also add the need to provide social media instruction for nonusers. Acceptability of the group was positive overall; however, it would likely be received more eagerly if there was communication between larger numbers of participants.

### Engagement

Most of the participants in this sample were in their 50s and 60s. Thus, alternative platforms need to be considered for engaging adults over the age of 60 years. Although Facebook use here in the United States was 73% for those aged 50-64 years in 2021 [31], it may be that participants in this sample did not find it useful for the Chat intervention. Other social media apps and platforms are not as popular as Facebook among older adults [31]; however, WhatsApp, which has end-to-end encryption, may improve privacy and increase trust for app-based interventions [32]. Currently, WhatsApp is popular among Hispanic American people, but its use is low among non-Hispanic Black and White people [31]. YouTube on the other hand is popular among a broad demographic and is the most used platform in the United States [31], making it a potential venue for future research.

Engagement may also be influenced by the availability and use of specific devices. Although smartphone, computer, and tablet ownership among older adults was less than 15% a decade ago, those numbers have been rapidly increasing [33]. Of those 65 years of age and older, 61% owned a smartphone in 2021 and 44% owned a tablet [33]. Regardless, several issues with engagement will need to be addressed for future studies. There might have been more activity in the Facebook group if the postings were more frequent and more personalized. One person commented that they would have liked notifications when new posts were added. Additionally, if each post must be approved by the IRB, it would be important to have a large number of articles and interesting videos for posting prior to the IRB application. One of the participants stated that the videos were helpful; therefore, more active and interactive content would likely be well received.

The feasibility of the intervention could not be determined for this study due to the small sample. As a result, future research using innovative recruitment techniques may be required before a determination of feasibility can be made. A future intervention should allow for freer communication, greater interaction, and include entertaining content. In addition, researchers should consider ways to personalize social media posts in order to improve engagement. Alternative platforms may be more acceptable and should be examined for a larger CR social media intervention. Finally, the length of the pre-post questionnaires should be considered moving forward. It may be that the questionnaires for this study were too burdensome, which is also evidenced by unanswered questions and missing data.

### Limitations

This study had limitations. The sample size was small, creating a high risk for type II error and a risk of bias related to unequal group sizes. The unequal group sizes resulted in unequal variances between groups, affecting the types of analyses that could be performed. Although comments in the Chat group were positive overall, given the nature of social media, it is possible that low participation rates in this study were due to privacy concerns. To address potential privacy concerns, alternative social media platforms should be considered. Furthermore, the sample was limited to 4 hospitals within a single organization. A more diverse population will be required for a larger study to improve generalizability. No between-group differences were found for any of the hypothesized outcomes since the study was underpowered. This was a feasibility study and a large sample size was not planned; however, a larger study will be required to determine whether the Chat intervention is feasible. Finally, “likes” were used as a surrogate measure of engagement; however, it is possible that participants valued a post and did not “like” it.

### Conclusions

Acceptability of the Facebook group was high for support and feeling in touch with providers. Despite challenges with recruitment and engagement, many important lessons were learned from this feasibility study. No between-group differences were found for motivational or need satisfaction scores. The small sample size likely affected the ability to find between-group differences and determine intervention feasibility. Nevertheless, among both groups, participants with greater exercise motivation at CR intake and greater improvement in perceived competence, completed more sessions, indicating that these are important factors for CR completion. More research is needed to find ways to engage those who have low motivation, and innovative recruitment methods may be required to ensure an adequate sample size. Researchers considering conducting social media research should consider various platforms for intervention delivery.

## Appendix

Multimedia Appendix 1 CONSORT-eHEALTH checklist (V 1.6.2).

## Abbreviations

BREQ-3: Behavioral Regulation in Exercise Questionnaire-3
Chat: Cardiac Rehabilitation Facebook Intervention
CR: cardiac rehabilitation
IRB: institutional review board
MI: myocardial infarction
PNSE: Psychological Need Satisfaction for Exercise
RAI: relative autonomy index

## Data Availability

The data sets generated and analyzed during this study are not publicly available due to institutional policy but are available from the corresponding author on reasonable request.
