# Supplementary material for: Cardiac Rehabilitation Facebook Intervention: Feasibility Randomized Controlled Trial
Source: JMIR Cardio. 2023 Jun 15;7:e46828. doi: 10.2196/46828 (PMC10334713; doi:10.2196/46828)
Supplement: Multimedia Appendix 1 [file cardio_v7i1e46828_app1.pdf]

CONSORT-EHEALTH Checklist V1.6.2 Report	Manuscript Number	46828

(based on CONSORT-EHEALTH V1.6), available at [http://tinyurl.com/consort-ehealth-v1-6].

Date completed
6/9/2023 13:20:25
by
Siegmund

Cardiac Rehabilitation Facebook Intervention: Feasibility Randomized Controlled Trial

TITLE

1a-i) Identify the mode of delivery in the title
"The purpose of this study was to determine the feasibility of the Cardiac Rehabilitation Facebook Intervention (Chat) intervention for affecting changes in exercise motivation and need satisfaction and adherence to cardiac rehabilitation."

1a-ii) Non-web-based components or important co-interventions in title

1a-iii) Primary condition or target group in the title
"Participants also qualified for the study if they were regular Facebook users (defined as logging in at least twice per month), had a cardiac diagnosis, and had been referred to the cardiac rehabilitation program at the hospital's main campus or 3 of the regional campuses."

ABSTRACT

1b-i) Key features/functionalities/components of the intervention and comparator in the METHODS section of the ABSTRACT
"The Behavioral Regulation in Exercise Questionnaire-3 and Psychological Need Satisfaction for Exercise were used to measure motivation and need satisfaction (competence, autonomy, and relatedness) before and after the Chat intervention. To support need satisfaction, the intervention included educational posts, supportive posts, and interaction with peers. The feasibility measures included recruitment, engagement, and acceptability. Groups were compared using analysis of variance and Kruskal-Wallis tests. Paired t tests were used to assess motivation and need satisfaction change, and Pearson or Spearman correlations were used for continuous variables."

1b-ii) Level of human involvement in the METHODS section of the ABSTRACT

1b-iii) Open vs. closed, web-based (self-assessment) vs. face-to-face assessments in the METHODS section of the ABSTRACT
"Recruitment took place during patients' inpatient stay, intake visit for cardiac rehabilitation, or via phone call within a week after hospital discharge."

1b-iv) RESULTS section in abstract must contain use data
"Thirty-two participants were lost to follow-up and 22 were included in the analysis."

1b-v) CONCLUSIONS/DISCUSSION in abstract for negative trials
"Acceptability of the Chat group was high; however, intervention feasibility could not be determined due to the small sample size"

INTRODUCTION

2a-i) Problem and the type of system/solution
"The primary purpose of this study was to determine the feasibility of the Chat Facebook intervention, providing education, peer support, and provider support, for affecting change in motivation and self-determination for exercise, and adherence to CR in patients with heart disease during a 12-week phase II CR program compared to a control group who received educational handouts and emails."

2a-ii) Scientific background, rationale: What is known about the (type of) system
"No Facebook interventions for cardiac rehabilitation adherence were found in the literature. Nevertheless, patients with greater Facebook capability were receptive to the idea of using the platform for a closed CR group and peer support."

Does your paper address CONSORT subitem 2b?
"We hypothesized that Chat would improve motivation for exercise, and participants in the Chat intervention would complete a higher percentage of CR sessions compared to the control. Additionally, we hypothesized that engagement in Chat would predict higher percentage of CR sessions and would provide evidence of feasibility. Feasibility was also determined by recruitment or sample size and retention."

METHODS

3a) CONSORT: Description of trial design (such as parallel, factorial) including allocation ratio
This was not described. It was a feasibility study.

3b) CONSORT: Important changes to methods after trial commencement (such as eligibility criteria), with reasons
No important changes were made.

3b-i) Bug fixes, Downtimes, Content Changes

4a) CONSORT: Eligibility criteria for participants
"planned to attend the outpatient CR", "were regular Facebook users", and "had a cardiac diagnosis, and had been referred to the cardiac rehabilitation program at the hospital's main campus or 3 of the regional campuses"

4a-i) Computer / Internet literacy
"were regular Facebook users "

4a-ii) Open vs. closed, web-based vs. face-to-face assessments:
"Recruitment took place during patients' inpatient stay, intake visit for cardiac rehabilitation, or via phone call within a week after hospital discharge."

4a-iii) Information giving during recruitment
"Written informed consent was obtained from each participant."

4b) CONSORT: Settings and locations where the data were collected
"the outpatient CR (center-based program) at the main campus and 3 regional hospitals of a large hospital system in Ohio"

4b-i) Report if outcomes were (self-)assessed through online questionnaires
"Change in motivation for exercise was measured using the BREQ-3, a 24-question instrument based on self-determination theory that measures intrinsic and extrinsic regulation of exercise behavior" and "Perception of psychological need satisfaction was measured with the PNSE to determine the extent to which participating in exercise promoted feelings of competence, autonomy, and relatedness, which are the 3 subscales of the PNSE"

4b-ii) Report how institutional affiliations are displayed

5) CONSORT: Describe the interventions for each group with sufficient details to allow replication, including how and when they were actually administered

5-i) Mention names, credential, affiliations of the developers, sponsors, and owners

5-ii) Describe the history/development process

5-iii) Revisions and updating

5-iv) Quality assurance methods

5-v) Ensure replicability by publishing the source code, and/or providing screenshots/screen-capture video, and/or providing flowcharts of the algorithms used

5-vi) Digital preservation

5-vii) Access
"A link was emailed to the participants and included the baseline Behavioral Regulation in Exercise Questionnaire-3 (BREQ-3) [28], Psychological Need Satisfaction for Exercise (PNSE) scale [25], and instructions for joining the private Chat group if applicable."

5-viii) Mode of delivery, features/functionalities/components of the intervention and comparator, and the theoretical framework
"The Cardiac Rehabilitation Facebook Intervention (Chat) was grounded in self-determination theory."

5-ix) Describe use parameters

5-x) Clarify the level of human involvement

5-xi) Report any prompts/reminders used
No reminders were used to prompt Facebook use; however, inclusion was regular Facebook use.

5-xii) Describe any co-interventions (incl. training/support)
No training was provided for the intervention.

6a) CONSORT: Completely defined pre-specified primary and secondary outcome measures, including how and when they were assessed

"The outcomes for this study were (1) change in motivation for exercise, (2) change in self-determination for exercise (competence, autonomy, and relatedness), (3) adherence to the 12-week CR program, and (4) measures of feasibility (recruitment strategy, success of the intervention, engagement, and acceptability)." "Engagement, defined as participation in the Chat group, was determined by the number of “likes” and “hits” in the group." "Acceptability of the intervention was determined from a post questionnaire, which had a section for additional comments." "Change in motivation for exercise was measured using the BREQ-3." "Perception of psychological need satisfaction was measured with the PNSE."

6a-i) Online questionnaires: describe if they were validated for online use and apply CHERRIES items to describe how the questionnaires were designed/deployed

6a-ii) Describe whether and how “use” (including intensity of use/dosage) was defined/measured/monitored

6a-iii) Describe whether, how, and when qualitative feedback from participants was obtained

6b) CONSORT: Any changes to trial outcomes after the trial commenced, with reasons

"the outpatient CR (center-based program) at the main campus and 3 regional hospitals of a large hospital system in Ohio"

7a) CONSORT: How sample size was determined

7a-i) Describe whether and how expected attrition was taken into account when calculating the sample size

7b) CONSORT: When applicable, explanation of any interim analyses and stopping guidelines

"The outcomes for this study were (1) change in motivation for exercise, (2) change in self-determination for exercise (competence, autonomy, and relatedness), (3) adherence to the 12-week CR program, and (4) measures of feasibility (recruitment strategy, success of the intervention, engagement, and acceptability)." "Engagement, defined as participation in the Chat group, was determined by the number of “likes” and “hits” in the group." "Acceptability of the intervention was determined from a post questionnaire, which had a section for additional comments." "Change in motivation for exercise was measured using the BREQ-3." "Perception of psychological need satisfaction was measured with the PNSE."

8a) CONSORT: Method used to generate the random allocation sequence

A random number generator was used.

8b) CONSORT: Type of randomisation; details of any restriction (such as blocking and block size)

"After the first 8, participants were randomized to the Chat versus the control groups using blocked randomization, an appointment was scheduled to discuss the study and obtain consent."

9) CONSORT: Mechanism used to implement the random allocation sequence (such as sequentially numbered containers), describing any steps taken to conceal the sequence until interventions were assigned

Sequentially numbered envelopes were used.

10) CONSORT: Who generated the random allocation sequence, who enrolled participants, and who assigned participants to interventions

The principal investigator generated the random allocation sequence, enrolled, and assigned.

11a) CONSORT: Blinding - If done, who was blinded after assignment to interventions (for example, participants, care providers, those assessing outcomes) and how

11a-i) Specify who was blinded, and who wasn’t

The statistician was blinded.

11a-ii) Discuss e.g., whether participants knew which intervention was the “intervention of interest” and which one was the “comparator”

11b) CONSORT: If relevant, description of the similarity of interventions

This is not relevant to this study

12a) CONSORT: Statistical methods used to compare groups for primary and secondary outcomes

"Categorical variables were described using frequencies and percentages, and analyses comparing the control and the Chat groups used Pearson chi-square or Fisher exact tests. Normally distributed continuous variables were described using means and SDs, and analyses comparing control and Chat groups used analysis of variance models. Non-normally distributed continuous variables were described using medians and quartiles, and analyses comparing the control and the Chat groups used Kruskal-Wallis tests. Paired t-tests were used to assess RAI and PNSE change within groups. The relationship between RAI change and continuous variables was assessed using Pearson or Spearman correlations (for the number of sessions which was not normally distributed) with 95% CI. For categorical measures, means and SDs with P-values from analysis of variance models. The relationships between the number of sessions with continuous variables were assessed using Spearman correlations with 95% CI, while medians and quartiles are presented for categorical factors. Internal consistency was determined with Cronbach α. Analyses were performed using SAS software (version 9.4; SAS Institute, Inc). A significance level of .05 was assumed for all tests."

12a-i) Imputation techniques to deal with attrition / missing values

Intention to treat analysis was used.

12b) CONSORT: Methods for additional analyses, such as subgroup analyses and adjusted analyses

No subgroup analyses were performed beyond previously stated.

RESULTS

13a) CONSORT: For each group, the numbers of participants who were randomly assigned, received intended treatment, and were analysed for the primary outcome

"Of 54 (26%) who agreed to take part in the study, 28 were randomized to intervention and 26 to the control group."

13b) CONSORT: For each group, losses and exclusions after randomisation, together with reasons

"Allocated to Control (n=26), Received control condition (n=20), Did not receive control condition-Hospital readmission (n=5), Moved out of state (n=1), Lost to Follow-up-Discontinued intervention/did not attend CR (n=12). Analyzed (n=8)."
"Allocated to intervention (n=28), Received allocated intervention (n=27), Did not receive allocated intervention-Hospital readmission (n=1). Lost to Follow-up-Discontinued intervention/did not attend CR (n=14)."

13b-i) Attrition diagram

14a) CONSORT: Dates defining the periods of recruitment and follow-up

Not in ms. Data collection started 3/2018. Follow-up ended 3/2019

14a-i) Indicate if critical “secular events” fell into the study period

14b) CONSORT: Why the trial ended or was stopped (early)

Trial ended after one year. Recruitment was a challenge.

15) CONSORT: A table showing baseline demographic and clinical characteristics for each group

Table 1. The relationship between group and demographic variables.
Overall (N=22)Control (N=8)Facebook (N=14)
Factor NStatisticsnStatisticsnStatisticsp-value
Age 2257.8±11.0860.6±7.21456.2±12.60.38a
Sex 22 8 14 0.19d
. M9(40.9) 5(62.5) 4(28.6)
. F13(59.1)3(37.5) 10(71.4)
Race 21 8 13 0.99d
. B 6(28.6) 2(25.0) 4(30.8)
. W 15(71.4) 6(75.0) 9(69.2)
RAI 22 39.4±23.5832.9±14.9 1443.1±27.00.34a
METS 16 5.9±2.4 66.5±2.2 105.6±2.6 0.52a
Diagnosis 22 8 140.079c
. CAD 14(63.6)7(87.5)7(50.0)
. other 8(36.4)1(12.5)7(50.0)
CR Sessions 22 26.0[4.0,36.0]822.0[3.5,27.0]1432.5[10.0,36.0]0.21b
Statistics presented as Mean ± SD, Median [P25, P75] or N (column %).
p-values: a=ANOVA, b=Kruskal-Wallis test, c=Pearson's chi-square test or d=Fisher's Exact test.

15-i) Report demographics associated with digital divide issues

n(%) for Age=57.8±11.0 and Gender=13(59.1) female. "Of the final analyzed sample, diagnoses included aortic aneurysm repair (n=2), myocardial infarction (MI) (n=7), coronary artery bypass graft without MI (n=1), percutaneous coronary intervention without MI (n=1), Takotsubo's cardiomyopathy (n=1), valve repair or replacement (n=3), heart transplant (n=2), and other (n=5). Of additional participant medical conditions, 64% had hypertension, 14% had diabetes, 14% had hypertriglyceridemia (>150 mg/dL), 32% had elevated low-density lipoprotein (≥100 mg/dL), and 45% had low high-density lipoprotein (<60 mg/dL). The mean functional capacity for participants at intake to CR was 5.9 (SD 2.4) METS. Data for exit metabolic equivalents were not analyzed as they were only available for 3 participants. Of the 14 remaining in the intervention group, 6 (43%) completed the exit questionnaires compared to 7 (87%) in the control group."		
**16a) CONSORT: For each group, number of participants (denominator) included in each analysis and whether the analysis was by original assigned groups**		
**16-i) Report multiple "denominators" and provide definitions**		
"Allocated to Control (n=26), Received control condition (n=20), Did not receive control condition-Hospital readmission (n=5), Moved out of state (n=1), Lost to Follow-up-Discontinued intervention/did not attend CR (n=12). Analyzed (n=8)."<br>"Allocated to intervention (n=28), Received allocated intervention (n=27), Did not receive allocated intervention-Hospital readmission (n=1). Lost to Follow-up-Discontinued intervention/did not attend CR (n=14)."<br>Final analyzed sample: Control=8; Intervention=14		
**16-ii) Primary analysis should be intent-to-treat**		
**17a) CONSORT: For each primary and secondary outcome, results for each group, and the estimated effect size and its precision (such as 95% confidence interval)**		
"Across both groups, higher motivation (RAI) at intake was associated (95% CI) with greater number of completed sessions [0.53 (0.14, 0.78), p=0.010]. Change in PNSE need satisfaction-autonomy from pre-to-post intervention was also associated with higher number of completed sessions [0.61 (0.09, 0.87), p=0.024]. No between group differences were found for change in motivation, need satisfaction, or sessions completed."		
**17a-i) Presentation of process outcomes such as metrics of use and intensity of use**		
"CR sessions out of 36, median (IQR)"		
**17b) CONSORT: For binary outcomes, presentation of both absolute and relative effect sizes is recommended**		
There were no binary outcomes.		
**18) CONSORT: Results of any other analyses performed, including subgroup analyses and adjusted analyses, distinguishing pre-specified from exploratory**		
There were no subgroup analyses.		
**18-i) Subgroup analysis of comparing only users**		
**19) CONSORT: All important harms or unintended effects in each group**		
No known unintended effects or harm took place.		
**19-i) Include privacy breaches, technical problems**		
**19-ii) Include qualitative feedback from participants or observations from staff/researchers**		
DISCUSSION		
**20) CONSORT: Trial limitations, addressing sources of potential bias, imprecision, multiplicity of analyses**		
**20-i) Typical limitations in ehealth trials**		
"The sample size was small, creating a high risk for type II error, and also a risk of bias related to unequal group sizes. The unequal group sizes resulted in unequal variances between groups, affecting the types of analyses that could be performed. Although comments in the Chat group were positive overall, given the nature of social media, it is possible that low participation rates in this study were due to privacy concerns."		
**21) CONSORT: Generalisability (external validity, applicability) of the trial findings**		
**21-i) Generalizability to other populations**		
**21-ii) Discuss if there were elements in the RCT that would be different in a routine application setting**		
**22) CONSORT: Interpretation consistent with results, balancing benefits and harms, and considering other relevant evidence**		
**22-i) Restate study questions and summarize the answers suggested by the data, starting with primary outcomes and process outcomes (use)**		
What is "the feasibility of the Chat Facebook intervention, providing education, peer support, and provider support, for affecting change in motivation and self-determination for exercise, and adherence to CR in patients with heart disease during a 12-week phase II CR program, compared to a control group who received educational handouts and emails?"		
**22-ii) Highlight unanswered new questions, suggest future research**		
Other information		
**23) CONSORT: Registration number and name of trial registry**		
ClinicalTrials.gov # NCT02971813		
**24) CONSORT: Where the full trial protocol can be accessed, if available**		
Journal of Medical Internet Research Protocols		
**25) CONSORT: Sources of funding and other support (such as supply of drugs), role of funders**		
Sigma Theta Tau International		
**X26-i) Comment on ethics committee approval**		
"This feasibility study was approved as minimal risk research by the hospital's institutional review board (IRB) (16-1456)"		
**x26-ii) Outline informed consent procedures**		
**X26-iii) Safety and security procedures**		
**X27-i) State the relation of the study team towards the system being evaluated**		
